# Identification of the Powdery Mildew Resistance in Chinese Wheat Cultivar Heng 4568 and its Evaluation in Marker-Assisted Selection

**DOI:** 10.3389/fgene.2022.819844

**Published:** 2022-02-17

**Authors:** Huiming Gao, Xiaozhe Xu, Pengfei Ai, Fuyi Luo, Peng Guo, Pengtao Ma

**Affiliations:** ^1^ College of Food Science and Biology, Hebei University of Science and Technology, Shijiazhuang, China; ^2^ College of Life Sciences, Yantai University, Yantai, China; ^3^ School of Computer and Control Engineering, Yantai University, Yantai, China; ^4^ Dezhou Agricultural Technology Extension and Seed Industry Center, Dezhou, China

**Keywords:** wheat powdery mildew, Heng 4568, molecular markers, MAS, *Pm2*

## Abstract

Powdery mildew induced by *Blumeria graminis* f. sp. *Tritici* (*Bgt*) has a devastating impact on global wheat yield and quality. Host resistance is the most effective and economical means to control this disease. In this study, Heng 4568, an elite wheat cultivar, shows high resistance to 12 *Bgt* isolates from different regions in China at the seedling stage. Genetic analysis demonstrates that the powdery mildew resistance in Heng 4568 is conferred by a single dominant locus, temporarily designated *PmH4568*. Furthermore, *PmH4568* is mapped to the reported *Pm2* interval on chromosome 5DS with five *Pm2* linked markers and flanked by the markers *Bwm20* and *Bwm21* with a genetic distance of 0.3 and 0.6 cM, respectively. To further investigate the relationship between *PmH4568* and *Pm2*, the diagnostic marker *Pm2b-map-3* of *Pm2* is used to genotype the F_2:3_ population derived from the cross Heng 4568 × Daimai 2173. Notably, there is no recombination found, indicating that *PmH4568* is also probably a *Pm2* allele. In addition, five closely linked markers as well as one diagnostic marker are successfully developed and tested in 16 wheat cultivars from different agro-ecological areas in China, which have potential applications in molecular breeding by marker-assisted selection.

## Introduction

Wheat powdery mildew incited by the biotrophic fungus *Blumeria graminis* f. sp. tritici (*Bgt*) is a foliar disease worldwide (Wu et al., 2019). The rapid spread of powdery mildew will cause severe wheat yield losses in a short time, especially in the winter wheat-growing regions with high inputs of irrigation and fertilizers (Luo et al., 2009; Hao et al., 2014). In China alone, the area of winter wheat affected annually by powdery mildew has exceeded 6 mha during recent decades, causing 300,000 tons of crop loss each year (http://cb.natesc.gov.cn/sites/cb/). With the climate getting warmer, the epidemics of wheat powdery mildew in China are growing more severe, which will always be a serious threat to national food security.

Given the significant yield-limiting effects of powdery mildew, the research and exploration of effective prevention and control technology has become urgent in wheat production. Currently, chemical control, biological control, and cultivation of disease-resistant varieties are common means. Chemical control is mainly by spraying fungicides to kill the *Bgt* isolates; however, it always pollutes the environment and accelerates variation of the *Bgt* isolates (Ma et al., 2018, 2019). Biological control mainly relies on some natural beneficial microorganisms and/or some existing substances in nature, to act as a natural antagonist of pathogen to resist other plant pathogen (Curtis et al., 2012). In comparison, host resistance is relatively the most effective, economical, and environmental way to control wheat powdery mildew, including broadening wheat resistance sources, polymerizing disease-resistant genes, and spreading disease-resistant cultivars (Felsenstein et al., 2010; Ma et al., 2018, Ma et al., 2019).

To date, more than 100 powdery mildew resistance (*Pm*) genes/alleles have been identified at 63 loci in wheat and its relatives (Li et al., 2019; McIntoshet al., 2019; He et al., 2021a; He et al., 2021b). Although several *Pm* genes have been widely used in production and provided high protection at both seedling and adult plant stages, more and more *Pm* genes are no longer effective against powdery mildew due to virulent mutants of the *Bgt* isolates (Xiao et al., 2013). Therefore, it is necessary to increase the genetic diversity of the resistant genes and characterize more effective alleles in wheat germplasms.

When a *Pm* gene was identified, its utilization efficiency in wheat production was mainly decided by its effectiveness and the agronomic performance of its donor (Zhao et al., 2013; Ma et al., 2018). There are some reports that several genes cannot be easily used for genetic improvement of powdery mildew resistance because of the linkage drag, competition drag, and adverse pleiotropism (Jia et al., 2020). One example is the gene *Pm16,* which is able to provide high resistance to different *Bgt* isolates, but will cause severe reduction of 15% in yield (Summers and Brown, 2013). Usually, commercial wheat cultivars have excellent agronomic performance without significantly bad traits and could be used as donors of valuable genes. In fact, several *Pm* genes have been identified from the cultivars with broad-spectrum resistance, such as *PmJM23* from Jimai 23 (Jia et al., 2020), *Pm52* from Liangxing 99 (Zhao et al., 2013), *PmW14* from Wennong 14 (Song et al., 2014), and *PmTm4* from Tangmai 4 (Xie et al., 2017). Therefore, characterization of powdery mildew resistance in the elite cultivars is important for isolating the underlying genes, which could be rationally used in breeding.

Marker-assisted selection (MAS) has enormous potential to improve the efficiency and precision in wheat breeding. Compared to the conventional breeding, MAS can combine several functional alleles from several individuals into one single genotype more precisely, with less unintentional losses and in fewer selection cycles (Xu and Crouch. 2008; Jiang et al., 2017). To perform MAS, closely linked molecular markers play a key role in tracing the targeted genes in the breeding population. Up to now, many molecular markers closely linked to *Pm* genes have been developed for MAS and efficiently used in different genetic backgrounds, thereby generating a large number of wheat breeding lines or resistant cultivars (Ma et al., 2018; Shah et al., 2018; Yu et al., 2018; Ye et al., 2019; Jia et al., 2020; Zhang et al., 2021). For instance, using tightly linked markers to *Pm2* and *Pm21* or co-segregate with *Pm4a*, three two-gene combinations, namely, *Pm2* + *Pm4a*, *Pm2* + *Pm21*, and *Pm4a* + *Pm21*, were successfully transferred into the commercial wheat cultivar “Yangmai 158” and double homozygotes were selected from the F_2_ population (Liu et al., 2000). In addition, MAS was also applied for other disease resistant in wheat, such as *Fusarium* head blight and stripe rust (Steiner et al., 2017; Nsabiyera et al., 2018; Su et al., 2018; Randhawa et al., 2019; Maré et al., 2020).

Heng 4568, an elite wheat cultivar, shows high resistance to powdery mildew. A previous study indicated that Heng 4568 most likely carries the known *Pm52* inherited from Liangxing 99 (Zou et al., 2017). Notably, Heng 4568 showed a broader resistant spectrum to different *Bgt* isolates than Liangxing 99 in our evaluation of disease resistance. Therefore, the objectives of this study include (1) analyzing its powdery mildew resistance using different *Bgt* isolates, (2) clarifying the presence of other *Pm* genes in Heng 4568 besides *Pm52*, and (3) developing molecular markers of the new identified *Pm* gene for MAS.

## Materials and Methods

### Plant Materials

The winter wheat cultivar Heng 4568 crossed by Hengyou 18 and Liangxing 99 was provided by the Institute of Dry Farming Agriculture, Hebei Academy of Agriculture and Forestry Sciences, and used as the donor of resistant gene(s) against powdery mildew. Wheat cultivar Daimai 2173 served as a susceptible parent that was crossed with Heng 4568 to produce F_1_ hybrids, F_2_ population, and F_2:3_ families for genetic analysis and molecular mapping of the *Pm* gene(s) in Heng 4568. Five wheat cultivars/lines with known *Pm* genes, namely, Liangxing99 (*Pm52*), Wennong14 (*PmW14*), Zhongmai155 (*PmZ155*), Jimai22 (*Pm52* + *PmJM23*), and Ulka/8*Cc (*Pm2a*), were used to compare their reaction patterns to different *Bgt* isolates with that of Heng 4568. Susceptible wheat cultivar Huixianhong was used as the susceptible control for phenotypic assessment. Sixteen susceptible wheat cultivars from different ecological regions in China (Hebei, Shandong, Henan, Shaanxi, Beijing, Anhui, and Jiangsu provinces in China) were used to evaluate the availability of closely linked markers for MAS.

### Assessment of Disease Resistance at the Seedling Stage

From 2019 to 2021, the assessment of disease resistance at the seedling stage was carried out in the greenhouse at Hebei University of Science and Technology (Shijiazhuang, China). Twelve *Bgt* isolates were collected from different wheat production regions in China. They were used to determine the reaction patterns of Heng 4568 and wheat genotypes with known *Pm* genes. For each *Bgt* isolate, at least 20 seeds for each genotype were sown in 128-cell rectangular trays in a growth chamber. The susceptible control Huixianhong was randomly planted in the tray. When the first leaves were unfolded, the seedlings were inoculated by *Bgt* conidiospores that were previously increased on Huixianhong seedlings. Then, the inoculated seedlings were incubated in an airtight dark environment for 24 h and then allowed disease symptom development in a greenhouse with a daily cycle of 14 h of light at 22°C and 10 h of darkness at 18°C (Qu et al., 2020). To perform genetic analysis, *Bgt* isolate KD07 was selected to inoculate seeding of Heng 4568, the susceptible cultivar Daimai 2173, and their F_1_, F_2_, and F_2:3_ progenies. For F_1_ hybrids, 10 plants were sown; for F_2_ population, 177 plants were sown; for F_2:3_ families, 172 families and 20–30 plants per family were sown. Two weeks after inoculation when the spores were fully developed on the susceptible controls, infection types (ITs) on the primary leaves of plants were rated with a scale of 0, 0;, 1, 2, 3, and 4. The leaves that displayed ITs 0–2 and 3–4 were regarded as resistant and susceptible, respectively (Liu et al., 1999). Three repeated experiments were carried out using the same procedure.

### Molecular Marker Analysis

Genomic DNA was extracted from the young leaf tissues following the cetyltriethylammonium bromide method (Saghai-Maroof et al., 1984). Resistant and susceptible DNA bulks were created by separately mixing equal amount of DNA from 10 homozygously resistant and 10 homozygously susceptible plants, respectively. Forty-eight molecular markers closely linked to 37 known *Pm* genes were firstly screened for their polymorphisms between Heng 4568, Daimai 2173, and their derived resistant and susceptible bulks (Supplementary Table S1). When the *Pm* gene in Heng 4568 was preliminarily to *Pm2* locus, other *Pm2* linked markers *Cfd81*, *Bwm20*, *Bwm21*, *Bwm25*, and *Swgi067* (Lu et al., 2015; Ma et al., 2018) and the diagnostic marker *Pm2b-map-3* (Jin et al., 2021) were also used to add the marker density for conducting the linkage map (Supplementary Table S2). PCR was performed in a 10-μl reaction volume containing 1 µl of 40–50 ng/μl template genomic DNA, 4.5 µl of 2×Taq Master Mix (Vazyme, China), and 0.5 µl of 10 μM/μl primer mix. The PCR program used was 95°C for 5 min; 36 cycles of 95°C for 30 s, 50–60°C (depending on specific primers) for 40 s, final extension at 72°C for 5 min; and storage at 4°C. PCR products were separated in 8.0% nondenaturing polyacrylamide gels with 29:1 ratio of acrylamide and bis-acrylamide with 1 × TBE buffer and then silver-stained and visualized as previously described (Santos et al., 1993).

### Statistical Analysis and Linkage Map Construction

After confirming genotypes of F_2:3_ families of Heng 4568 and Daimai 2173, the deviations of the observed phenotypic data from theoretically expected segregation ratios for goodness of fit were assessed using *χ* ^2^ test. MAPMAKER 3.0 and the Kosambi function were performed to construct the linkage map of the powdery mildew resistance gene in Heng 4568.

## Results

### Evaluation of Powdery Mildew Resistance in Heng 4568

Heng 4568 was highly resistant to 12 *Bgt* isolates with the ITs 0–2, whereas Daimai 2173 and susceptible control Huixianhong were all highly susceptible to all the tested *Bgt* isolates (Table 1). Compared with the *Pm52* donor Liangxing 99, Heng 4568 was resistant to the *Bgt* isolates KD03, KD07, KD08, and KD11, while *Pm52* was susceptible to these four Bgt isolates, indicating that Heng 4568 contains other *Pm* gene(s).

**TABLE 1:** Infection types of Heng 4568 and other genotypes with *Pm2* alleles to 12 *Blumeria graminis* f. *sp. tritici* (*Bgt*) isolates at the seedling stage.

Cultivar/lines	Pm genes	*Blumeria graminis tritici* isolates (*Bgt*)											
		KD01	KD02	KD03	KD04	KD05	KD06	KD07	KD08	KD09	KD10	KD11	KD12
Heng 4568	*PmH4568 + Pm52*	0	0	1	0	0	0	1	1	0	0	2	0
Ulka/8*Cc	*Pm2a*	0	0	3	0	0	1	4	3	0	0	4	0
Liangxing 99	*Pm52*	0	0	4	0	0	0	4	4	0	0	4	0
Wennong 14	*PmW14*	0	0	2	0	0	0	3	3	0	0	3	0
Zhongmai 155	*PmZ155*	0	0	2	0	0	0	2	3	0	0	3	0
Jimai 22	*PmJM23+Pm52*	0	0	1	0	0	0	0	0	0	0	2	0
Daimai 2173	*-*	4	4	4	4	4	4	4	4	3	4	4	3
Mingxian 169	*-*	4	4	3	4	4	4	4	4	3	4	4	4

Note: A 0–4 scale was used to scored infection types (IT), of which 0, 0; 1 and 2 are considered to be resistant, while those with an IT score of 3 or 4 are considered to be susceptible. Wheat genotypes Huixianhong and Daimai 2173 were used as susceptible controls.

### Genetic Analysis of *Pm* Genes in Heng 4568

To explore other *Pm* gene(s) besides *Pm52* in Heng 4568, the isolate KD07 virulent to Liangxing 99 (with *Pm52*) and avirulent to Heng 4568 was selected to inoculate Heng 4568, Daimai 2173, and their derived F_1_ seeds, F_2_ population, and F_2:3_ families, respectively. All the tested F_1_ seedlings were resistant to KD07 similar to their parent Heng 4568. The F_2_ population fitted the segregation ratio of a single dominant gene (Table 2). The harvested F_2:3_ families from the F_2_ population confirmed the expected ratio of 1:2:1 (Table 2). Therefore, it was concluded that another dominant *Pm* gene is also involved in Heng 4568, which was temporarily designated *PmH4568*.

**TABLE 2:** Segregation ratios of F_2_ and F_2:3_ generations of Heng 4568 × Daimai 2173 following inoculation with *Blumeria graminis* f. s. *tritici* (*Bgt*) isolate KD07 at the seedling stage.

Cross	Plants observed			Expected ratio	*χ* ^2^	*p*
	HR	Seg	HS			
Heng4568 × Daimai2173 F_2_	130		47	3:1	0.23	0.63
Heng4568 × Daimai2173 F_2:3_	43	83	46	1:2:1	0.31	0.85

Note: Values of *χ* ^2^ for statistical significance at *p* = 0.05 are 3.84 (1df) and 5.99 (2df); HR, homozygous resistant, Seg: segregating, HS, homozygous susceptible. Discrepancies on the line numbers between F2 and F3 generation are because several F2 plants were died during the growth process.

### Molecular Mapping of *PmH4568*

**FIGURE 1:**
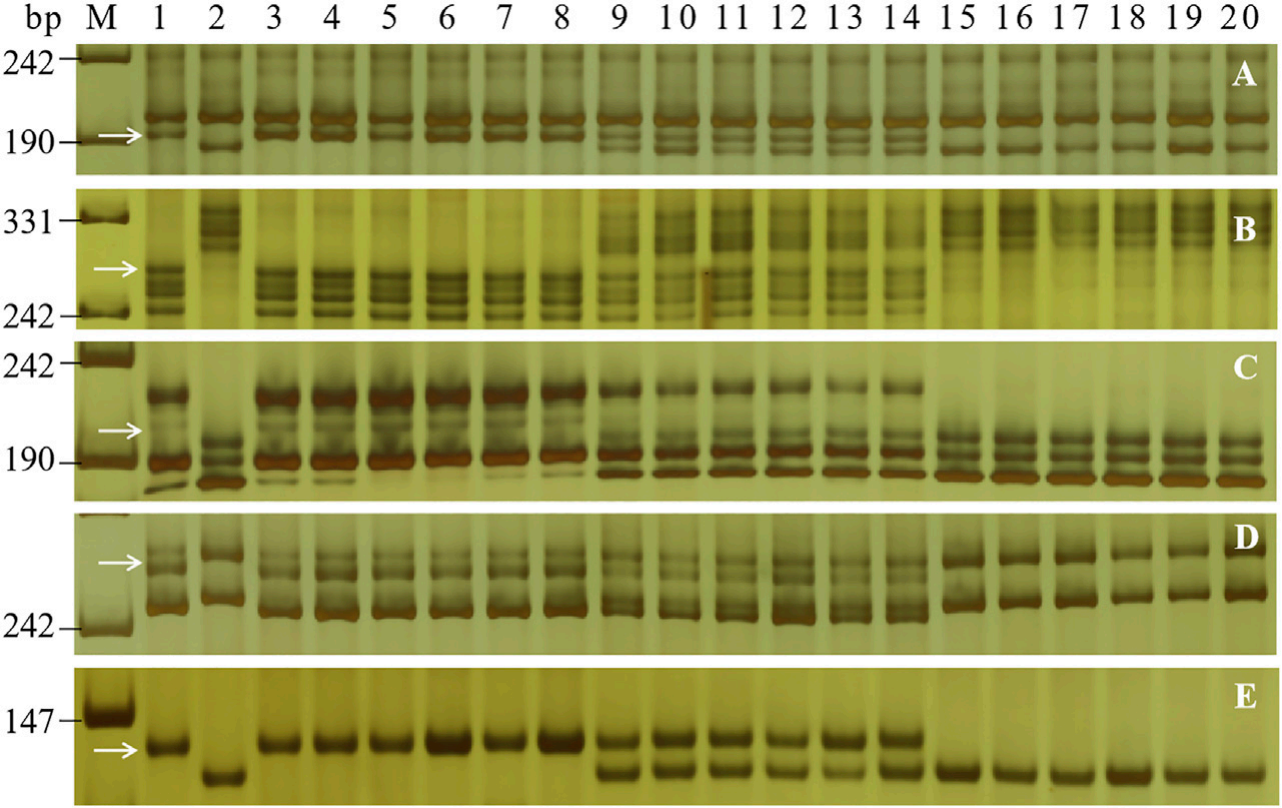
PCR amplification patterns of the selected markers *Bwm20* **(A)**, *Bwm21* **(B)**, *Bwm25* **(C)**, *Cfd81* **(D)**, and the diagnostic *Pm2b-map-3* **(E)** in genotyping Heng 4568, Daimai 2173, and random selected F_2:3_ families of Heng 4568 × Daimai 2173. Lane M: pUC19 *Msp* I; lanes 1–2: Heng4568 and Daimai 2173; lanes 3–8: homozygous-resistant F_2:3_ families; lanes 9–14: heterozygous F_2:3_ families; lanes 15–20, homozygous susceptible F_2:3_ families. The white arrows indicate the polymorphic bands in Heng4568.

**FIGURE 2:**
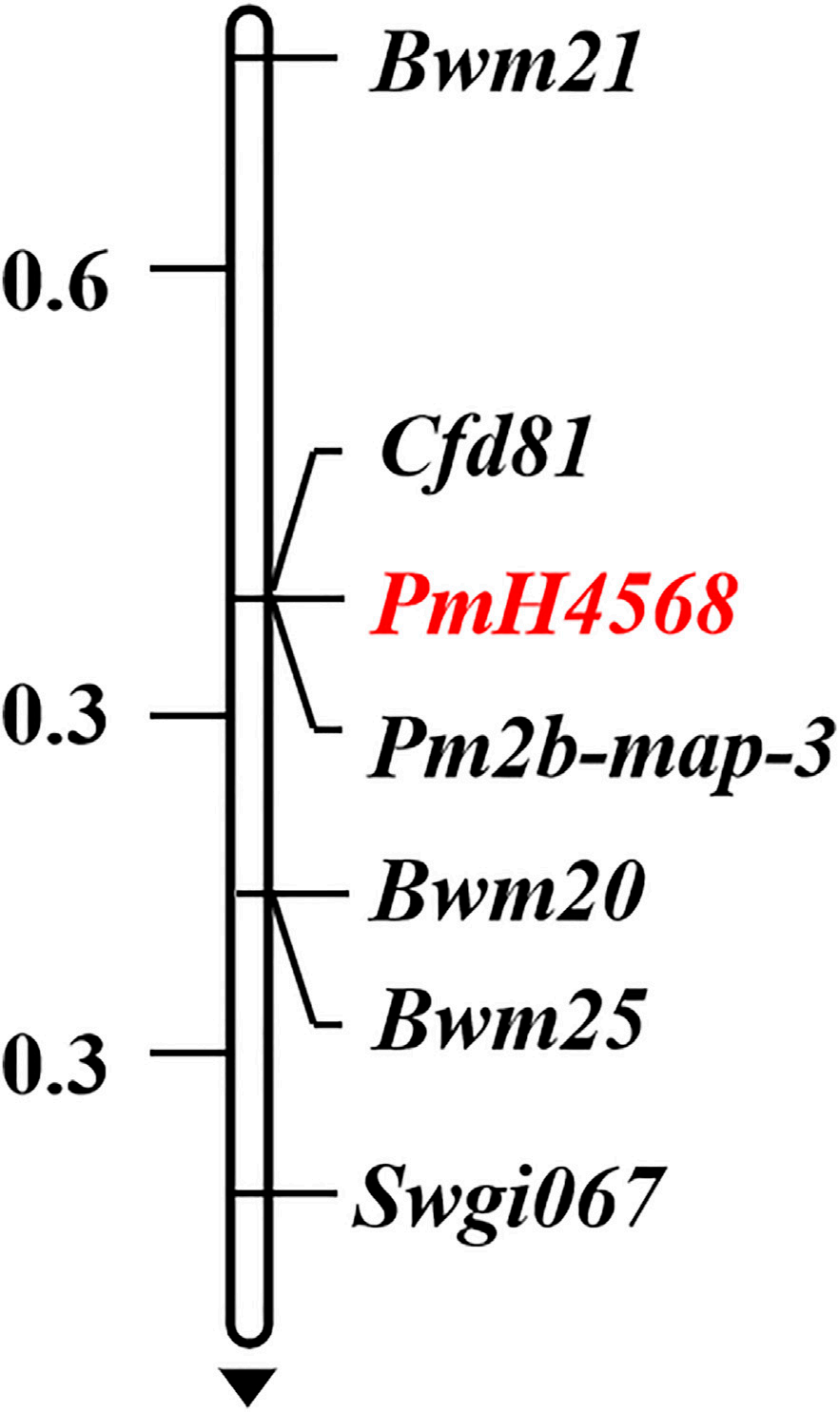
Linkage map of *PmH4568* using the F_2:3_ families of Heng 4568 × Daimai 2173. Genetic distances in cM are showed to the left. The black arrow points to the centromere.

To determine the genetic location of *PmH4568*, 48 molecular markers closely linked to the known *Pm* genes were firstly used to test their polymorphisms between the parents and the two contrasting bulks. The *Pm2*-linked marker *Cfd81* showed consistent polymorphism between Heng 4568, Daimai 2173, and their derived contrasting bulks. Then, *Cfd81* was used to genotype the F_2:3_ families of Heng 4568 and Daimai 2173 and confirmed its linkage relationship with *PmH4568* (Figures 1, 2; Supplementary Table S2). This suggested that *PmH4568* was most likely located in the *Pm2* interval. To confirm this interval, four additional *Pm2*-linked markers, *Bwm20*, *Bwm21*, *Bwm25*, and *Swgi067*, were also proved to be closely linked to *PmH4568* (Figure 1; Supplementary Table S2). A genetic linkage map was then conducted to locate *PmH4568* to the *Pm2* interval (Figure 2). To further confirm the relationship between *PmH4568* and *Pm2*, *Pm2b-map-3*, the diagnostic marker of *Pm2*, was used to genotype the F_2:3_ families of Heng4568 and Daimai 2173 (Figure 1; Supplementary Table S2). No recombinants were found, suggesting that *PmH4568* was located in the *Pm2* locus and most likely a *Pm2* allele.

### Evaluation of Closely Linked Markers for Marker-Assisted Selection

**FIGURE 3:**
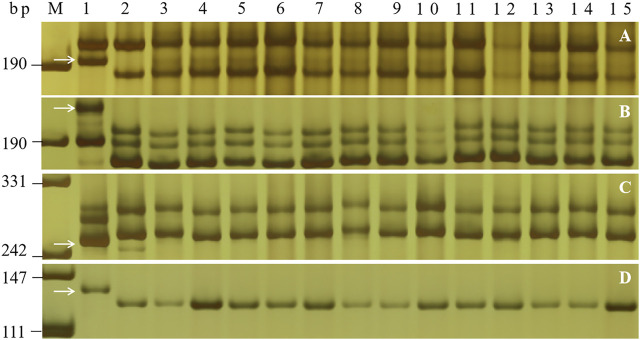
PCR amplification patterns of the markers *Bwm20* **(A)**, *Bwm25* **(B)**, *Cfd81* **(C)**, and *Pm2b-map-3* **(D)** in genotyping Heng 4568, Daimai 2173, and 13 selected wheat cultivars. Lanes M: pUC19 *Msp* I; lanes 1–2: Heng 4568 and Daimai 2173; lanes 3–15: Shannong 1538, Hanmai 13, Zhoumai 27, Xinong 979, Jimai 229, Jimai 21, Jimai 20, Zhongyu 9398, Womai 8, Shimai 15, Xinluo 4, Zhengmai 0856, and Wunong 6. The white arrows indicate the polymorphic bands in Heng 4568.

To transfer *PmH4568* to susceptible cultivars using MAS, five *PmH4568*-linked markers, *Bwm20*, *Bwm21*, *Bwm25*, *Swgi067*, and *Cfd81*, and the diagnostic marker *Pm2b-map-3*, were used to test Heng 4568 and 16 susceptible cultivars. The results showed that all the tested markers could amplify polymorphic bands between Heng 4568 and these susceptible cultivars, indicating that once *PmH4568* is transferred into the susceptible cultivars through conventional hybridization, these markers can be used to detect *PmH4568*, especially the diagnostic marker *Pm2b-map-3* (Figure 3; Table 3).

**TABLE 3:** Validation of PmH4568-linked and diagnostic markers on 16 Chinese wheat cultivars in marker-assisted selection (MAS) breeding.

Cultivars	Region	*Bwm20*	*Bwm21*	*Bwm25*	*SWGI067*	*Cfd81*	*Pm2b-map-3*
Heng 4568	Hebei	+	+	+	+	+	+
Daimai 2173	Shandong	-	-	-	-	-	-
Shannong 1538	Shandong	-	-	-	-	-	-
Hanmai 13	Hebei	-	-	-	-	-	-
Zhoumai 27	Henan	-	-	-	-	-	-
Xinong 979	Shaanxi	-	-	-	-	-	-
Jimai 229	Shandong	-	-	-	-	-	-
Jimai 21	Shandong	-	-	-	-	-	-
Jimai 20	Shandong	-	-	-	-	-	-
Zhongyu 9398	Henan	-	-	-	-	-	-
Womai 8	Anhui	-	-	-	-	-	-
Shimai 15	Hebei	-	-	-	-	-	-
Xinluo 4	Henan	-	-	-	-	-	-
Zhengmai 0856	Henan	-	-	-	-	-	-
Wunong 6	Shaanxi	-	-	-	-	-	-
Huaimai 0226	Jiangsu	-	-	-	-	-	-
Luchen 185	Shandong	-	-	-	-	-	-
Zhongyu 1311	Beijing	-	-	-	-	-	-

Note: “+” represents that the markers cannot amplify the polymorphic products linked to *PmH4568* in the tested genetic backgrounds, and “-” shows the opposite result.

## Discussion

Heng 4568 is an elite winter wheat cultivar in Northern China. Due to its superior agronomic performance and powdery mildew resistance, Heng 4568 is considered as an attractive cultivar and serves as a favorable breeding parent for resistance improvement. A previous study indicated that the known *Pm52* located on the chromosome 2BL was involved in Heng 4568, which may confer the powdery mildew resistance (Zou et al., 2017). *Pm52* is a widely used *Pm* gene in Chinese cultivars, such as Hanong 2312, Zhongxinmai 99, Shimai 26, and DH51302. Heng 4568 was derived from the cross of Liangxing 99 with Hengyou 18, indicating that *Pm52* in Heng 4568 may be derived from its parent Liangxing 99. However, our study demonstrated that Heng 4568 showed significantly broader resistant spectrum than the *Pm52* donor Liangxing 99, suggesting that other *Pm* genes may also be involved in Heng 4568. To clarify the composition of the *Pm* genes in Heng 4568, a *Bgt* isolate virulent KD07 was used to identify other *Pm* gene(s) in Heng 4568. The result showed that another dominant *Pm* gene *PmH4568* also contributed to the powdery mildew resistance in Heng 4568. This result, together with a previous study, provided an explicit genetic constitution for the powdery mildew resistance in Heng 4568, which contributes to scientific parental selection collocation.

Using *Pm2*-linked markers, *PmH4568* was mapped to the known *Pm2* interval on chromosome arm 5DS. According to previous studies, a series of *Pm2* alleles have been reported in the *Pm2* interval, such as *Pm2a* (Qiu et al., 2006), *Pm2b* (Ma et al., 2015a), *Pm2c* (Xu et al., 2015), *PmLX66* (Huang et al., 2012), *PmX3986-2* (Ma et al., 2014), *PmWFJ* (Ma et al., 2015b), *PmYB* (Ma et al., 2015c), *PmZ155* (Sun H. et al., 2015), *PmW14* (Sun Y. et al., 2015), *PmWFJ* (Ma et al., 2015a), *PmSub* (Jin et al., 2018), *Pm10V-2* (Ma et al., 2018), *PmJM23* (Jia et al., 2020), and *PmFG* (Ma et al., 2016)*.* Using MutChromSeq (Mutant chromosome sequencing) (Sáchez-Martín et al., 2016) and analysis of the fine mapping interval (Chen et al., 2019), *Pm2* was cloned and confirmed to encode a CC-NBS-LRR protein. Haplotype analysis of 48 hexaploid common wheat carrying *Pm2* alleles showed that all these *Pm2* donors have the perfectly consistent as the cloned sequence above. However, different *Pm2* alleles derived from hexaploid common wheat have significantly different resistant spectra (Ma et al., 2018; Jia et al., 2020). This may be due to their different genetic backgrounds, and also complex genetic constitution or resistance mechanism may be involved in this interval. Anyway, *Pm2* is an elite gene locus that is very valuable for resistance breeding, even though the complex *Pm2* locus has not been fully characterized.

In wheat resistance breeding using MAS, the breeding potential of a certain gene depends not only on its resistance but also on the comprehensively agronomic traits, such as yield, quality, and high combining ability. Thus, although many *Pm* genes/alleles have been identified, only several have been widely used in breeding programs (Li et al., 2011). The main obstacle that limits the application of these genes is linkage drag in most resistance donors. After transferring these *Pm* genes into susceptible commercial cultivars, unfavorable traits linked to them will lead to poor agricultural yield or quality performances (Ma et al., 2015b). Therefore, the resistance donors with excellent agricultural performance are very popular for breeders. Fortunately, Heng 4568 is a cultivar with desirable comprehensively agronomic traits. For powdery mildew resistance, Heng 4568 also carries *Pm52* besides the *Pm2* allele *PmH4568*. In China, *Pm2* and *Pm52* are two major *Pm* genes in many resistance cultivars, such as Liangxing 66, Wennong 14, YingBo 700, Zhongmai 155, Jimai 23 (Jia et al., 2020), and Nongda 399, which all have the *Pm2* allele, and Liangxing 99, Hannong 2312, Zhongxinmai 99, DH51302, and Zhimai 26, which all have *Pm52* (Qu et al., 2020). Compared with these resistance cultivars, Heng 4568 has two resistance genes, which may show more durable resistance than a single resistance gene; such a situation also involved Jimai 22, a famous cultivar with the largest promotion area in the last 10 years (Jia et al., 2020). For the yield and quality, there was no significant defect in the recent years in our field. Particularly worth mentioning is its high combining ability in breeding; two famous wheat cultivars, Hengmai 28 and Jiamai 361, have been released in production using Heng 4568 as parent (https://www.Chinaseed114.com/seed/16/seed_77094.html; https://www.chinaseed114.com/seed/14/seed_69187.html), and in our lab, Heng 4568 is also a popular breeding parent for both resistance and yield improvement. Therefore, Heng 4568 can be not only directly popularized in region with high incidence of powdery mildew, but also used as a valuable breeding parent to improve powdery mildew resistance.

To transfer the *Pm* genes in Heng 4568, MAS is a rapid and effective way (William et al., 2007; Ashra and Foolad, 2013; Collard and Mackill, 2018). Since fine mapping of *Pm52* has been carried out, many closely linked markers of *Pm52* have been developed for MAS (Wu et al., 2019). In this study, the applicability of five closely linked markers and one diagnostic marker has been investigated in MAS with 16 susceptible wheat cultivars. In particular, the diagnostic marker *Pm2b-map-3* is a functional marker designed by SNPs within the *Pm2* sequence, suggesting that there is no recombinant in MAS. Therefore, resistance breeding using Heng 4568 as a parent is promising, and more *trans*-breeding studies using Heng 4568 are under way in our lab.

## Conclusion

In this study, *PmH4568*, an effective *Pm* gene in the elite cultivar Heng 4568, has been identified and proved to be a *Pm2* allele. We further clarify the genetic components of the powdery mildew resistance in Heng 4568. The applicability of closely linked markers, including the diagnostic marker, was validated in MAS. Overall, this work will accelerate the utilization of the powdery mildew resistance in Heng 4568.

## Data Availability

The original contributions presented in the study are included in the article/Supplementary Material, further inquiries can be directed to the corresponding author.
